# Septic arthritis in neonatal and juvenile New World camelids (5 cases)

**DOI:** 10.1093/jvimsj/aalag056

**Published:** 2026-03-25

**Authors:** Mitja Miklavcic, Isabelle Kilcoyne, Julie E Dechant, Bridget Ratliff, K Gary Magdesian

**Affiliations:** William R. Prichard Veterinary Medical Teaching Hospital, UC Davis School of Veterinary Medicine, Davis, CA, United States; Department of Surgical and Radiological Sciences, UC Davis School of Veterinary Medicine, Davis, CA, United States; Department of Surgical and Radiological Sciences, UC Davis School of Veterinary Medicine, Davis, CA, United States; Comstock Equine Hospital, Reno, NV, United States; Department of Medicine and Epidemiology, UC Davis School of Veterinary Medicine, Davis, CA, United States

**Keywords:** alpaca, joint, llama, sepsis

## Abstract

Septic arthritis (SA) in camelids is infrequently reported in the literature. The purpose of this study was to describe the clinical presentation, etiologies, diagnostics, treatment and short- and long-term outcome in neonatal and juvenile New World camelids (NWCs) with SA. Two neonatal alpaca crias were diagnosed with SA associated with failure of transfer of passive immunity and neonatal sepsis. Three juvenile camelids, 1 alpaca, and 2 llamas were presented with an infection of a single joint with associated lameness. All animals were treated with systemic and intra-articular antimicrobials. Surgical treatment including joint lavage was performed in 4 cases. Four (80%) NWCs survived to discharge. Three were sound at the time of discharge and 1 was discharged with mild lameness. One neonatal cria was euthanized. Septic arthritis in neonatal and juvenile NWC appears to have a favorable prognosis for discharge and guarded prognosis for long-term outcome with appropriate treatment.

## Introduction

Septic arthritis (SA) represents an important disease in livestock species with high morbidity and death rate.^1–3^ While etiologies of SA in equids are reported,^4^ there is a paucity in the literature regarding this condition in camelids, with relatively few case reports.^5,6^

Early intervention is imperative and treatment includes systemic and local antimicrobial therapy.^7^ Surgical intervention, including lavage of the affected joints with arthroscopy or needle lavage, is an effective adjunctive therapy to systemic antimicrobials in other species.^8–12^ Survival is reported as fair to good in foals (78%-93%)^10,11^ and guarded to fair long term in calves (59% of 54).^13^ Factors associated with lower survival in foals included involvement of multiple synovial structures and concurrent disease.^11^

Septic arthritis has been described infrequently in New World camelids (NWCs), and information pertaining to diagnosis, treatment, and prognosis in these species is lacking.^5^ The objectives of this case series are to describe signalment, history, clinicopathological data, imaging findings, treatment, and outcome in neonatal and juvenile NWC with SA.

## Methods

The criteria for inclusion in this study were a diagnosis of SA in neonatal or juvenile NWCs based on clinical signs and diagnostic imaging findings, in conjunction with cytological analysis of synovial fluid confirming a total nucleated cell count of ≥ 25 000 cells/μL, with ≥ 80% neutrophils, total protein concentration ≥ 4.5 g/dL, and the presence of intracellular bacteria on cytology, or a positive bacterial culture of synovial fluid.^13^ Animals were excluded if no synovial fluid analysis was performed, or the medical record was incomplete.

Medical records from the William R. Pritchard Veterinary Medical Teaching Hospital at the University of California-Davis School of Veterinary Medicine for the years 2000-2022 were reviewed. Animals were divided into 2 groups based on age: neonatal (≤1 month old) and juvenile (1-12 months).

All animals underwent physical and orthopedic examination. Diagnostics included synoviocentesis and subsequent cytological analysis and culture. Radiographic and ultrasonographic imaging, or ultrasonographic imaging, were also pursued. Data retrieved from the records included signalment, history, clinicopathological data, synovial fluid cytological analysis and culture results, ultrasonographic and radiographic findings, inciting cause (if known), treatment, and duration of hospitalization. Failure of transfer of passive immunity (FTPI) was defined as serum (IgG) concentration < 900 mg/dL or immunocrit < 11%.^14–16^ All antimicrobial breakpoints were extrapolated from CLSI (Clinical and Laboratory Standards Institute) equine guidelines or published pharmacokinetic studies in alpacas and are applicable only to parenteral antibiotics. Interpretation for orally administered antimicrobials were not included because the bioavailability and disposition of these drugs administered in camelids is unknown.^17–19^

Short-term survival was defined as survival to hospital discharge. Long-term survival was defined as survival for ≥ 6 months after hospital discharge. Long-term follow-up was obtained through use of medical records or telephone questionnaire with the owners.

### Treatment

Through and through needle lavage was performed in the sedated or anesthetized animal with sterile polyionic fluids using 14 or 16G needles. Ultrasonographic guidance was used to facilitate placement in select cases. All joints were medicated with antimicrobials at the end of lavage. Arthrotomy, when pursued, was performed by making 2 incisions followed by removal of purulent material and thorough lavage of the exposed joint using sterile polyionic fluids.

Systemic antimicrobial use was recorded, in addition to any analgesic medications administered. In cases where intravenous regional limb perfusion (IVRLP) was performed, a tourniquet was applied proximal to the affected joint and the perfusate was administered slowly into the cephalic vein. The tourniquet was left in place for 20 min.

## Results

Five camelids were included in the study. These included 2 llamas, a 5-week-old female and 12-month-old male, and 3 alpacas, a 4-day-old female, a 4-month-old female, and a 6-day-old male. Affected joints included the radiocarpal (*n* = 1), coxofemoral (*n* = 1), lateral metacarpophalangeal (*n* = 1), and elbow (*n* = 2) joints. Two cases (cases 1 and 2) were diagnosed with concurrent FTPI and septicemia (Table S1).

### History and clinical presentation

Two neonatal crias (cases 1 and 2) were presented for lethargy and fever. Case 1 had been diagnosed with FTPI and had been treated with procaine penicillin G (PPG) before presentation. The cria was obtunded, tachycardic (140 bpm; reference range 70-100 bpm^6^) and febrile on presentation (39.4°C; reference range; < 38.9°C^6^). Septic arthritis was suspected based on heat and swelling on palpation of the left carpus. Case 2 was presented with lameness associated with the left hind limb and had been treated with PPG before presentation. The cria presented with tachycardia (160 bpm), was normothermic, and was lame on the left hind limb with no discernable swelling.

Cases 3–5 were presented with normal vital signs with the primary presenting complaint of lameness. The degree of lameness varied from non-weight bearing in case 3, noticeable at the walk in case 4, and noticeable only at the trot in case 5. None of these cases had a history of trauma and no wounds were appreciated during initial examination. Swelling and heat was palpated over the respective lateral metacarpophalangeal (case 3) and elbow joints (cases 4 and 5). Case 4 had received intra-articular amikacin before presentation.

### Presenting clinicopathological data

A CBC was performed in all cases on admission (Table S1). The total white blood cell count (WBC) was low in case 1 (1890; reference 7890-20 270 cells/μL) and was within normal limits in the other cases (median 12 870; range 1890-17 580 cells/μL). The neutrophil count was low in case 1 (56; reference 3670-10 960 cells/μL) and high in case 4 (17 580; reference 3670-10 960 cells/μL), with a median of 9846 cells/μL (range 56-11 033 cells/μL). The median band neutrophil count across all cases was 64 cells/μL (range 0-1891 cells/μL). Fibrinogen was increased in all 5 cases (median 600; range 500-900 mg/dL; reference 100-300 mg/dL). Immunocrit was measured as 4% in case 2.

Blood cultures were submitted in both neonatal crias. Multiple organisms were cultured for case 1 including *Klebsiella* spp., *Enterobacter* spp., and Pseudomonas aeruginosa. A single organism (hemolytic Escherichia coli) was cultured in case 2.

### Joint fluid analysis and culture

Synoviocentesis was performed in all cases (Table S2). Ultrasonographic guidance was used in cases 1-4. In case 1, the low volume of obtained fluid permitted only cytological evaluation of a smear and bacterial culture. For case 2, the volume collected permitted bacterial culture only. The total protein concentration (*n* = 4: median 5.35; range 4.1-6.5 g/dL), total nucleated cell count (*n* = 3: range > 100 000 L—208 770/μL) and neutrophil percentage (*n* = 4: median 89.5%; range 80%-99%) were increased in all samples. In cases 1 and 3, neutrophils were predominantly degenerative, whereas case 4 was characterized by nondegenerate to mildly degenerative neutrophils, and case 5 by nondegenerate neutrophils. Bacteria were not identified on cytological examination of the synovial fluid in any case.

Synovial fluid was submitted for bacterial culture and sensitivity testing in all cases, of which 2 (cases 1 and 2) yielded bacterial growth. Organisms cultured matched those of the blood culture results and included *P aeruginosa* and *Klebsiella* spp. in case 1 and hemolytic *E coli* in case 2.

### Diagnostic imaging findings

Radiographs of the affected joint were taken in all cases. The images revealed soft tissue swelling (moderate *n* = 2 [cases 1 and 3] and severe *n* = 1 [case 4]), gas shadowing within the joint (*n* = 2; cases 2 and 4), evidence of physitis adjacent to the joint (*n* = 2; cases 1 and 3), osteomyelitis of subchondral bone of proximal left radius (*n* = 1; case 5), widening/enlargement of the joint (*n* = 2; cases 1 and 2) and mild osteoproliferation of the metaphysis (*n* = 1; case 4).

Ultrasonographic examination was performed in 4 cases (cases 1, 3, 4, and 5) and revealed joint effusion (mild *n* = 2 [cases 1 and 5], severe *n* = 1 [case 3]), gas shadowing within the joint (*n* = 1 [case 4]), severe synovial thickening in all affected joints and cortical bone irregularity adjacent to the affected joint (*n* = 2 [cases 3 and 5]).

### Treatment

All camelids were initially treated with broad spectrum systemic antimicrobial drugs and non-steroidal anti-inflammatory agents (NSAIDs) (Tables S3 and S4 and Item S1). Cases 1 and 2 were treated for FTPI and septicemia (Item S1).

The initial antibiotic selection was appropriate based on the susceptibility profiles of the isolated bacteria in cases where a positive culture was obtained. Antibiotic selection was changed for 1 neonatal cria (case 1) based on a poor response to initial therapy and clinician preference. Four cases (cases 2-5) were discharged on different antibiotics (Table S3).

A through and through needle lavage of the affected joint was performed under general anesthesia in 2 cases (cases 2 and 4) and under sedation in 1 additional case (case 3). Joint lavage was repeated 6 days later in case 3. An arthrotomy was performed 4 days after the initial joint lavage in case 4. All cases had intra-articular antimicrobials administered into the affected joint at the completion of the procedure (Table S3). Cases underwent a median of 2 intra-articular medication installations (range 1-4). One case (case 3) underwent a single intravenous regional limb perfusion using 50 mg ceftiofur sodium injected into the cephalic vein as an adjunctive treatment for septic physitis.

Cases 1 and 5 underwent intra-articular medication of the affected joint without further intervention.

### Follow-up and outcome

Length of hospitalization ranged from 4 to 10 days. Four of the 5 cases (80%) (cases 2-5) survived to discharge. Case 1 developed skin necrosis over the affected joint, which precluded additional synoviocentesis and intraarticular medication, and was subsequently euthanized on day 8 of hospitalization due to failure to respond to treatment. Necropsy was not performed. One animal (case 2) was mildly lame at the time of discharge but had significantly improved since presentation. Long-term follow-up was available for cases 2 and 5. Case 2 was still mildly lame 6 months after discharge and in case 5 the lameness resolved completely.

## Discussion

This case series documents a favorable short-term, and guarded long-term prognosis, for NWC with SA, with 80% (4/5) of cases surviving to discharge and 3/5 remaining sound. While SA has been cited as a possible sequalae of sepsis or FTPI in neonatal or juvenile camelids,^6^ it is infrequently reported in the literature. One study looking at clinical findings and survival in 56 ill neonatal NWC documented no cases with SA or umbilical infection.^20^ A more recent paper comparing clinical findings and prognostic outcome variables in critically ill neonatal foals and crias documented a much lower incidence of SA in neonatal crias with only 4/256 (1.6%) crias having SA, compared to 42/356 (11.5%) of foals.^21^ The reason for this difference is unknown, but could reflect differences in immune responses to sepsis or the microanatomy of the synovial vasculature between the 2 species.

In the current study, both neonatal crias were diagnosed with sepsis secondary to FPT and SA had likely occurred with hematogenous spread. The 3 juvenile cases presented for a solitary joint infection, with no concurrent systemic signs. Hematogenous infection was considered the most likely source in those cases, however, no previous infectious process was noted in the history. One case had concurrent osteomyelitis (case 5) identified on radiographic examination and one had evidence of septic physitis (case 3).

Bacterial culture of the synovial fluid in both neonatal crias yielded positive growth and matched the bacteria isolated on blood culture in both cases. *Klebsiella* spp. and *E coli* are reported from the septic neonatal NWC, however, to the authors’ knowledge *Enterobacter* spp. and *P aeruginosa* have not.^20,22,23^ Blood cultures on both neonatal crias in the current study only yielded growth of gram negative bacteria, in contrast to previous studies reporting 45%-80% gram positive isolates in critically ill neonatal crias.^20,22^ The difference could be attributed to the previous treatment with penicillin in our cases, the small number of cases, or might reflect geographically different populations of bacteria.

Radiographic examinations were performed in all cases, while ultrasonographic examinations were performed in 4 cases included in this study, with findings apparent in all cases. Osseous abnormalities, including the presence of osteomyelitis and septic physitis, were noted on radiographic examination in 3 cases. Radiography can, however, be of limited value for diagnosing infection of the bone in the first 7-10 days and osseous involvement should not be ruled out negative radiographic findings during that time frame. Ultrasonography can be a more sensitive indicator of boney irregularities early in the disease process; in the current study, it was helpful to identify the presence of thickened synovium and the gas within the joint, both indicative of SA. In addition, ultrasonography was instrumental in facilitating accurate needle placement for lavage and intra-articular instillation of antibiotics.

The treatment of SA in these cases followed the same principles extrapolated from other large animal species and included broad spectrum systemic antimicrobial therapy, in addition to local/regional antibiotic administration and lavage of the affected joint.^8,24^

Intravenous regional limb perfusion was performed in 1 case and is reported in the literature.^25^ No adverse effects were noted associated with the procedure and in appropriate cases IVRLP should be considered as a potential adjunct in treating SA in camelids.

All cases in this study had only one affected joint. Previous studies reporting on SA in foals have documented up to 23% involving more than 1 infected joint.^13,26^ Polyarthritis was documented in 26.5% affected calves, but found the median number of joints affected per animal to be lower than that reported for foals in other studies.^27,28^ Differences between species might reflect differences in clinical presentation, particularly regarding duration, immune response, or severity of bacteremia.^13^ It could also reflect anatomical differences between species and the ability of bacteria to localize and establish infection in multiple locations. The source of infection also differs, as reported by Bedenice et al., with significantly fewer neonatal camelids presented with umbilical conditions compared to their equine counterparts.^11,21^

Two of 5 NWCs in the current study presented with infection associated with the elbow joint, with 1 each having a septic radiocarpal joint, lateral metacarpophalangeal joint and coxofemoral joint. Previous studies have documented that SA is most commonly associated with the stifle and tarsal joints in foals.^10^ Similar findings are reported in calves presenting with SA, with 1 study reporting the carpal joints were the most frequently affected joints followed by the stifle and tarsus.^13^ One study did document SA of the cubital joint in 11/95 (12%) foals.^10^ It has been previously reported that larger joints might be at increased risk for bacterial colonization due to the larger surface area of synovium present.^13,29^ Anatomical differences between species also likely reflects the different joints affected.

The main limitations of this study include those inherent to its retrospective design. The limited number of cases over a long-time period might have contributed to variability in how cases were diagnosed and treated. In addition, reliance of the accuracy of medical records could have limited the retrieval of data.

In conclusion, results of this study indicate that SA in neonate and juvenile NWC does occur and most likely results as a sequalae to hematogenous bacterial spread. Treatment strategies extrapolated from other large animal species can be effective.

## Supplementary Material

aalag056_supplclean

## Off-label antimicrobial declaration

Amikacin, ampicillin, cefazolin, ceftiofur, florfenicol, oxytetracycline, and ticarcillin–clavulanate used off-label.

## Institutional animal care and use committee or other approval declaration

The authors declare no institutional animal care and use committee or other approval was needed.

## Abbreviations

CRI: constant rate infusion
FPT: failure of passive transfer
IgG: immunoglobulin G
IVRLP: intravenous regional limb perfusion
NWC: New World camelid
PPG: procaine penicillin G
SA: septic arthritis
WBC: white blood cell
