# Supplementary material for: Septic arthritis in neonatal and juvenile New World camelids (5 cases)
Source: J Vet Intern Med. 2026 Mar 25;40(2):aalag056. doi: 10.1093/jvimsj/aalag056 (PMC13018769; doi:10.1093/jvimsj/aalag056)
Supplement: aalag056_supplclean [file aalag056_supplclean.docx]

Additional diagnostics:

Both neonatal crias diagnosed with sepsis (Case 1 and 2) underwent additional diagnostic imaging consisting of abdominal and thoracic ultrasonography. Evidence of mild pneumonia was noted in both crias based on the presence of B lines and intestinal hypomotility was noted in Case 2.

Additional treatments:

Case 1 and 2 were treated for FPT and septicemia which included administration of a plasma transfusion (1-2 units (300ml) of llama plasma and additional supportive care consisting of intravenous fluid support, gastroprotectants (pantoprazole; 1mg/kg IV q24h), NSAIDs (flunixin 1mg/kg IV or IM, q12h or meloxicam 0.5mg/kg IV q48h), dextrose (4mg/kg/min CRI), insulin (0.01IU/kg/hr CRI), and thiamine (10 mg/kg IV diluted in fluids, q 12 h).

Cases 3-5 received nonsteroidal anti-inflammatory agents (flunixin meglumine 0.5mg/kg IV or meloxicam 1mg/kg PO) in addition to systemic antibiotics (Table 2). Case 3 and 4 were also administered pantoprazole (1mg/kg IV, q24h). The treatments were tapered as the clinical condition improved.

Tables:

| Case number | Species | Age | Joint affected | Systemic TNCC (cells/μL) | Neutrophils (cells/μL | Bands (cells/μL) | Fibrinogen (mg/dL) | Blood Culture | Antibiotic administered prior to presentation |  |
| --- | --- | --- | --- | --- | --- | --- | --- | --- | --- | --- |
|  |  |  |  |  |  |  |  |  |  |  |
| 1 | Alpaca | 4 days | Left radiocarpal | 1890 | 56 | 7 | 600 | *Klebsiella* spp, | Yes |  |
|  |  |  |  |  |  |  |  | *Enterobacter* spp and |  |  |
|  |  |  |  |  |  |  |  | *Pseudomonas aeruginosa* |  |  |
|  |  |  |  |  |  |  |  |  |  |  |
| 2 | Alpaca | 6 days | Left coxofemoral | 11820 | 6265 | 1891 | 800 | Hemolytic *E. coli* | Yes |  |
| 3 | Alpaca | 4 months | Left font lateral metacarpo | 12870 | 9846 | 64 | 900 |  | No |  |
|  |  |  | phalangeal |  |  |  |  |  |  |  |
| 4 | Llama | 5 weeks | Left elbow | 17580 | 11030 | 345 | 600 |  | Yes |  |
| 5 | Llama | 1 year | Left elbow | 13160 | 10133 | 0 | 500 |  | No |  |

Table 1: Signalment, affected joint, initial clinicopathological parameters, and previous treatments with antibiotics of the camelids included in the study. TNCC = total nucleated cell count.

| Case # | TNCC (cells/µL) | % Neutrophils | Total Protein (g/dL) | Bacteria Cultured |
| --- | --- | --- | --- | --- |
| 1 | n | 80 | 4.1 | *Pseudomonas aeruginosa, Klebsiella pneumoniae* |
| 2 | n | n | n | Hemolytic *Escherichia coli* |
| 3 | >100,000 | 98 | 6.5 | — |
| 4 | 165,200 | 81 | 5.2 | — |
| 5 | 208,770 | 99 | 5.5 | — |

Table 2: Results of the synovial fluid analysis and synovial bacterial species isolated. TNCC = total nucleated cell count.

| Case # | Antibiotics Used | Length of Systemic Antibiotic Tx | Intra-articular Antibiotics | Repeat Joint Lavage | Days of hospitalization |
| --- | --- | --- | --- | --- | --- |
| 1 | Ceftiofur sodium (5 mg/kg IV BID) + Amikacin (25 mg/kg IV SID) × 6 days → Amikacin + Ticarcillin clavulanate (50 mg/kg IV QID) | 8 days – until euthanasia | Amikacin 25 mg | N/A | 8* |
| 2 | Amikacin (22 mg/kg IV SID) + Ampicillin (30 mg/kg IV QID) × 5 days → Ceftiofur sodium (4.4 mg/kg IV BID) | 25 days | Ampicillin (unknown dose), Amikacin 100 mg | No | 10 |
| 3 | Ampicillin (20 mg/kg IV TID) + Amikacin (18 mg/kg IV SID) × 7 days → discharged on Ceftiofur sodium (5 mg/kg SC BID × 3 days then Ceftiofur crystalline free acid 5 mg/kg SC q3d) | Unknown | Amikacin 25 mg, Ceftiofur 25 mg | Yes | 8 |
| 4 | Ceftiofur sodium (2.2 mg/kg IV BID) + Florfenicol (20 mg/kg SC EOD) → discharged on Ceftiofur sodium (2.2 mg/kg IV BID) | 8 days (continued by referring veterinarian) | Not given | Yes | 7 |
| 5 | Oxytetracycline (20 mg/kg SC q3d) | 30 days | Cefazolin 1 g | N/A | 4 |

Table 3: Summary of the antibiotics used in the treatment of the septic arthritis, length of treatment and hospitalization. IV = intravenous, SC = subcutaneous, SID = once daily, BID = twice daily, TID = three times daily, QID = four times daily, EOD = every other day. * the cria was euthanized on day 8.

|  | Case 1 | | | | | Case 2 | |
| --- | --- | --- | --- | --- | --- | --- | --- |
|  | Synovial fluid | | Blood | | | Synovial fluid | Blood |
| **Antimicrobial** | ***Klebsiella pneumoniae*** | ***Pseudomonas aeruginosa*** | ***Klebsiella pneumoniae*** | ***Enterobacter asburiae*** | ***Pseudomonas aeruginosa*** | ***E coli*** | ***E coli*** |
| Amikacin | 1 (S) | 2 (S) | 1 (S) | 1 (S) | 2 (S) | 4 (S) | 1 (S) |
| Ampicillin | >16 (R) | >16 (R) | >16 (R) | 8 (R) | >16 (R) | 2 (S) | 2 (S) |
| Cefazolin | ≤2 (S) | >16 (R) | ≤2 (S) | >16 (R) | >16 (R) | ≤2 (S) | ≤2 (S) |
| Cefotaxime | ≤0.5 (S) | 16 (I) | ≤0.5 (S) | ≤0.5 (S) | 16 (I) | ≤0.5 (S) | ≤0.5 (S) |
| Ceftazidime |  |  |  |  |  | ≤4 (S) | ≤4 (S) |
| Cefpodoxime | ≤2 | >8 | ≤2 | ≤2 | >8 |  |  |
| Ceftiofur | 0.5 (S at higher doses)^+^ | >4 (NI) | 0.5 (S at higher doses)^+^ | 1 (S at higher doses)^+^ | >4 (NI) | ≤0.25 (S) | ≤0.25 (S) |
| Chloramphenicol | 4 | >16 (R) | 4 | 4 | >16 (R) | 4 | 4 |
| Doxycycline  (SC administration only) ^19^ | 4 (R) | >8 (R) | 4 (R) | 2 (R) | >8 (R) | 1 (R) | 1 (R) |
| Enrofloxacin | ≤0.25* (S) | 1 (R) | ≤0.25 (S) | ≤0.25* (S) | 1 (R) | ≤0.12 (S) | ≤0.12 (S) |
| Gentamicin | ≤0.25 (S) | 1 (S) | ≤0.25 (S) | ≤0.25 (S) | 1 (S) | 0.5 (S) | ≤0.25 (S) |
| Imipenem | ≤1 (S) | 2 (S) | ≤1 (S) | 2 (S) | 2 (S) | ≤1 (S) | ≤1(S) |
| Minocycline |  |  |  |  |  | 0.5 | 0.5 |
| Penicillin | >8 (R) | — |  |  | — |  |  |
| Ticarcilin + Clavulanate | ≤8 (S) | 16 (S) | ≤8 (S) | ≤8 (S) | 16 (S) |  |  |

Table 4: Minimum inhibitory concentrations (MICs, µg/mL) of antimicrobial agents for bacterial isolates obtained from synovial fluid and blood. S= Susceptible, I = Intermediate, R= Resistant are included for parenteral antimicrobials.

*Enrofloxacin: CLSI guidelines for enrofloxacin in horses has changed; whereas previously ≤0.25 µg/mL was considered susceptible, but current guidelines have lowered that to ≤0.12 µg/mL. In alpacas, a dose of 5 mg/kg SC, or 10 mg/kg PO, is appropriate for bacteria with MIC values of ≤0.5 µg/mL and ≤0.25 µg/mL, respectively. ^18^

+Ceftiofur: In extrapolating from horses, 0.25 µg/mL is considered the susceptibility breakpoint using labeled dosing (2 mg/kg IM, q 24 h). ^17^ Llamas achieve this concentration for 24 hours after 2.75 mg/kg IM once daily, and alpacas are expected to do so with 1.5 mg/kg IM or IV, q 12 h. ^17^ However, 0.5 and 1 µg/mL concentrations are achievable at higher (extra-label doses) for horses, which could be the case for camelids as well but requires further study.

SC = subcutaneous
